# Multi-Filter Quantum Neural Networks for Efficient Channel Estimation in RIS-Assisted Systems

**DOI:** 10.3390/s26134249

**Published:** 2026-07-04

**Authors:** Min-Hyeok Choi, Ja-Eun Kim, Seung-Han Kim, Myung-Sun Baek, Gyeong-Ho Lee, Duck-Dong Hwang, Hyoung-Kyu Song

**Affiliations:** 1 Department of Information and Communication Engineering, Sejong University, Seoul 05006, Republic of Korea; alsgurkk@sju.ac.kr (M.-H.C.); thinkdana@sju.ac.kr (J.-E.K.); 2 Department of Convergence Engineering for Intelligent Drone, Sejong University, Seoul 05006, Republic of Korea; justin5929@sju.ac.kr; 3 Department of AI Convergence Electronics Engineering, Sejong University, Seoul 05006, Republic of Korea; duckdonh@sejong.ac.kr; 4 Department of Artificial Intelligence and Information Technology, Sejong University, Seoul 05006, Republic of Korea; msbaek@sejong.ac.kr (M.-S.B.); gyeongho@sejong.ac.kr (G.-H.L.)

**Keywords:** reconfigurable intelligent surface, cascaded channel estimation, quantum convolutional neural network, quantum machine learning, multi-filter architecture, B5G/6G wireless communications, quantum-aided networking

## Abstract

A reconfigurable intelligent surface (RIS) is a promising technology for beyond-fifth-generation (B5G) and sixth-generation (6G) wireless communications, but its passive reflection and two-hop double-fading structure make cascaded channel estimation challenging. Conventional convolutional neural network (CNN) estimators require many trainable parameters, while a single shallow parameterized quantum circuit (PQC) may have limited feature representation. Deep quantum circuits can also suffer from noise and barren-plateau effects on noisy intermediate-scale quantum (NISQ) devices. To address these issues, this paper proposes a multi-filter quantum convolutional neural network (MF-QCNN) for cascaded channel estimation in RIS-assisted multi-user uplink systems. The proposed model uses multiple independent shallow PQC filters in parallel, concatenates their measured features, and estimates the cascaded channel through a compact classical dense head, with the total trainable-parameter count scaling as 182F+696 for *F* parallel filters. Simulation results, compared with a single-filter quantum convolutional neural network (QCNN), CNN, and multilayer perceptron (MLP) baselines, show that at a signal-to-noise ratio (SNR) of 20 dB, the 3-filter MF-QCNN reduces the normalized mean squared error (NMSE) by approximately 22.9, 8.1, and 4.6 dB relative to the single-filter QCNN, CNN, and MLP baselines, respectively, while using only about 19.3% of the CNN trainable parameters. Under zero-forcing (ZF) precoding, it achieves the highest achievable sum rate among the learning-based estimators; at SNR = 30 dB, it improves the achievable sum rate by approximately 17.4% and 12.8% over the CNN and MLP baselines, respectively. These simulation results suggest that the parallel shallow-PQC design can serve as a compact quantum-aided estimator for RIS channel estimation and may provide a useful basis for future studies on AI-native transceiver design in B5G/6G networks.

## 1. Introduction

Beyond-fifth-generation (B5G) and sixth-generation (6G) wireless communications are expected to support ultra-high data rates, massive connectivity, extremely low latency, and improved energy efficiency for emerging services such as extended reality, autonomous systems, and large-scale Internet of Things applications [1,2,3]. Reconfigurable intelligent surfaces (RISs) are regarded as key enablers for future 6G-oriented wireless networks because they can construct favorable radio environments by controlling the reflection response of many low-cost passive elements [4,5,6,7]. By creating a controllable reflected path, an RIS can help maintain coverage when the direct user–base station (BS) link is blocked.

RIS-assisted communication, however, requires accurate channel acquisition. Since a passive RIS generally has no sensing, data-processing, or baseband capability, the RIS-related channels cannot be directly estimated at the surface and are typically inferred indirectly at the BS [8,9,10]. Recent studies have also shown that channel training remains a central issue for hybrid and beyond-diagonal intelligent-surface architectures, where improved sensing capability or inter-element connectivity can reduce some bottlenecks but introduces new estimation-design tradeoffs [11,12]. This problem becomes more difficult when the direct user–BS link is severely blocked. In that case, data transmission mainly depends on the RIS-reflected path.

The effective cascaded channel is determined by the user–RIS channel, the RIS phase-shift matrix, and the RIS–BS channel. This creates a two-hop double-fading structure. Compared with a conventional single-hop channel, the cascaded channel is harder to reconstruct from noisy pilot observations. The reconstruction burden also increases as RIS-assisted systems scale in the numbers of users, BS antennas, and RIS reflecting elements. Therefore, B5G/6G-oriented RIS-assisted systems require channel estimators that are accurate, compact, and suitable for real-time resource-aware implementation.

This paper focuses on cascaded channel estimation in a RIS-assisted multi-user uplink system. The goal is to recover the effective cascaded channel from a pilot-processed noisy observation at the BS. Instead of estimating the individual RIS–user and RIS–BS links separately, the proposed estimator directly reconstructs the effective cascaded channel used for communication and precoder design.

### 1.1. Related Work

Several model-based channel estimation methods have been developed for RIS-assisted wireless systems. Wang et al. proposed a channel estimation framework for RIS-assisted multi-user communications based on structured pilot observations [13]. Their method reduces part of the channel acquisition burden, but its performance depends on pilot design and channel-structure assumptions. Wei et al. studied channel estimation for RIS-assisted multi-user multiple-input single-output (MISO) communications [14]. Their method improves multi-user channel acquisition, but the training overhead can increase as the system dimension grows. Chen et al. exploited common block sparsity for multi-user millimeter-wave (mmWave) multiple-input multiple-output (MIMO) channel estimation [15], while Li et al. used statistical channel state information (CSI) of correlated RIS–user channels to reduce pilot overhead in multi-user settings [16]. Tensor-based and super-resolution approaches have further been investigated for RIS-assisted full-duplex MIMO and multi-user mmWave MISO channel recovery [17,18]. These methods provide strong structural priors, but they may be sensitive to sparsity, correlation, array response, or training model assumptions.

Learning-based channel estimation has also been studied for RIS-assisted systems. Liu et al. proposed a deep denoising neural network with compressive channel estimation for mmWave intelligent reflecting surfaces [19]. This approach improves reconstruction from noisy measurements, but it still depends on a compressive estimation pipeline and sparsity assumptions. Convolutional neural network (CNN)-related denoising and reconstruction networks have also been investigated for RIS or mmWave channel estimation [20]. These studies demonstrate the effectiveness of classical deep-learning architectures for RIS channel estimation while also highlighting their dependence on classical feature extraction and reconstruction pipelines. Recent studies have further combined deep learning with joint cascaded-channel estimation and detection, masked-autoencoder-aided pilot allocation, and residual-attention reconstruction for RIS-assisted mmWave or multi-user MISO systems [21,22,23]. These results show the usefulness of learning-based estimators, but conventional neural-network estimators may still suffer from dimension-dependent parameter growth as the numbers of users, BS antennas, or RIS reflecting elements increase. Classical model-compression techniques offer one possible remedy for this parameter growth: network pruning removes redundant weights [24], and quantization reduces numerical precision for efficient inference [25]. These techniques are useful, but they still operate in the classical feature space, and they may trade accuracy for compression when the channel mapping is highly nonlinear, motivating an exploration of nonclassical feature-extraction strategies such as quantum machine learning (QML).

QML provides another direction for compact nonlinear feature extraction. QML models encode classical data into quantum states and process them through parameterized quantum circuits (PQCs) [26,27,28]. Quantum convolutional neural networks (QCNNs) combine quantum convolution and pooling operations in compact circuit architectures [29]. Quanvolutional models have also demonstrated the use of small quantum circuits for local feature extraction in image-recognition tasks [30]. More broadly, hybrid quantum–classical frameworks, resource-efficient QCNN structures, next-G QML surveys, and remote-sensing-oriented hybrid QCNNs have further been examined [31,32,33,34,35]. In wireless communications specifically, quantum-aided learning has recently been explored for variational-quantum-circuit-based turbo detection in coded MIMO systems and multiuser MISO downlink beamforming optimization [36,37]. In particular, Zhang et al. evaluated hybrid quantum–classical beamforming networks against a classical neural-network benchmark, providing a relevant comparison framework between quantum-aided and classical learning architectures [37]. Taken together, these studies establish the general feasibility of QML and of combining quantum and classical processing and show that variational quantum circuits can be embedded into physical-layer processing; however, the wireless-domain applications mainly address turbo detection and beamforming rather than cascaded channel estimation in RIS-assisted systems.

The general comparison methodology adopted in this paper, namely benchmarking a QCNN-type architecture against classical neural-network counterparts under matched training conditions, follows the precedent set by Hur et al., who found that QCNN models could match or exceed CNN classification accuracy on image-classification benchmarks while using substantially fewer trainable parameters [38]. These comparisons suggest that classical neural-network counterparts provide useful reference points for assessing quantum-aided models in terms of accuracy and parameter efficiency.

Among these QML directions, several QCNN-related studies are particularly relevant to the proposed architecture. Henderson et al.’s quanvolutional neural network uses multiple small quantum circuits for image-feature extraction, but its quantum filters are randomly initialized and fixed during training [30]. Song et al.’s resource-efficient QCNN improves the computational efficiency of a quantum convolutional layer, while Long et al.’s quantum–classical–quantum convolutional neural network (QCQ-CNN) adopts a serial quantum–classical–quantum pipeline [34,35]. However, these studies mainly focus on image-classification tasks and do not consider cascaded channel estimation in RIS-assisted wireless systems.

Increasing the depth of a single QCNN is one possible way to improve its expressiveness. However, in noisy intermediate-scale quantum (NISQ)-oriented settings, deeper PQCs may be more affected by gate noise and barren plateaus, which can limit reliable circuit execution and trainability [39,40,41]. These considerations motivate compact quantum-aided estimators that avoid unnecessary circuit-depth growth while maintaining sufficient representation capability for RIS-assisted cascaded channel recovery.

### 1.2. Main Contributions

To address these limitations, this paper proposes a multi-filter quantum convolutional neural network (MF-QCNN) for cascaded channel estimation in RIS-assisted multi-user uplink systems. The proposed architecture uses multiple shallow PQC filters in parallel. Each filter processes the same embedded channel observation and extracts a distinct quantum feature vector. The feature vectors are concatenated and decoded by a compact classical dense head. This parallel-filter structure expands the quantum feature representation along the filter dimension while keeping each PQC filter shallow.

The main contributions of this paper are summarized as follows.

- We formulate RIS-assisted uplink pilot processing as an effective cascaded-channel reconstruction problem. The BS directly estimates the cascaded channel from a pilot-processed noisy observation, without separately reconstructing the RIS–user and RIS–BS channels.
- We propose an MF-QCNN architecture for compact channel estimation. The model expands the quantum feature representation along the filter dimension while keeping each individual PQC filter shallow.
- We evaluate the proposed MF-QCNN using normalized mean squared error (NMSE), trainable-parameter count, and achievable sum rate under zero-forcing (ZF) precoding. The proposed method is compared with classical CNN and multilayer perceptron (MLP) baselines and a single-filter QCNN.
- We analyze the effect of the number of parallel PQC filters. The largest performance gain occurs in the transition from a single filter to two filters, while additional filters provide smaller gains. This result gives a practical guideline for selecting the filter count under limited quantum-resource budgets, supporting resource-aware deployment in B5G/6G networks.

The remainder of this paper is organized as follows. Section 2 presents the RIS-assisted system model, the cascaded channel model, and the pilot-based channel estimation problem. Section 3 describes the learning-based estimation framework, the QCNN, the proposed MF-QCNN, and the classical CNN and MLP baselines. Section 4 provides the evaluation settings, and Section 5 presents the numerical results. Section 6 discusses the practicality, novelty, applicability, and limitations of the proposed approach, together with directions for future work. Finally, Section 7 summarizes the findings.

## 2. System Model

### 2.1. RIS-Assisted Uplink System

This paper considers a RIS-assisted multi-user uplink system. The system comprises a multi-antenna BS equipped with $N_{t}$ antennas, *K* single-antenna users that transmit uplink pilot signals, and a RIS composed of *N* low-cost passive reflecting elements mounted on the facade of a nearby building.

**Figure 1:**
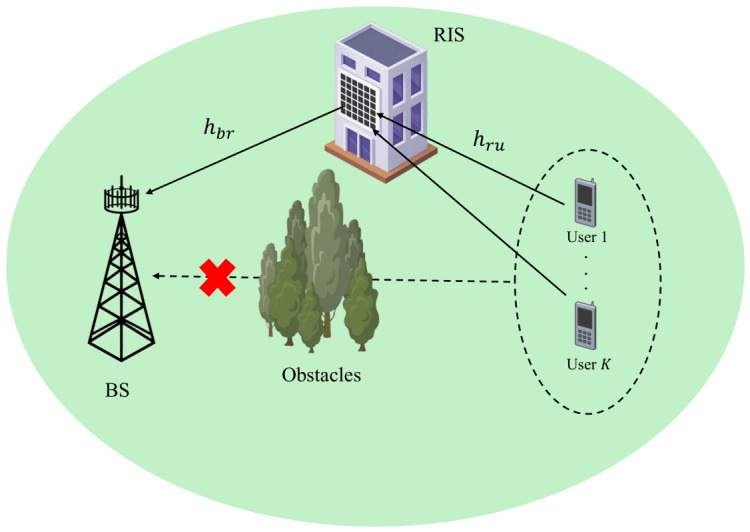
Illustration of the RIS-assisted multi-user uplink system model.

As illustrated in Figure 1, the direct propagation paths between the users and the BS are severely blocked by obstacles, such as high-rise buildings and dense foliage. As a result, reliable communication cannot be established through the direct line-of-sight link. To address this blockage, a RIS is deployed as a nearly passive reflecting surface that provides a controllable reflected path between the users and the BS.

We denote the BS–RIS link by $H_{br}\in C^{N\times N_{t}}$ and the RIS–user link by $H_{ru}\in C^{K\times N}$. Under channel reciprocity, these link coefficients are used to describe the corresponding reverse propagation paths during uplink pilot training. During uplink pilot transmission, the signals emitted by the users propagate over the RIS–user link, are reflected by the RIS, and then propagate over the BS–RIS link before being received by the BS antenna array.

The reflection behavior of the RIS is characterized by the diagonal phase-shift matrix

(1) $$\Phi =\mathrm{diag}\left(\phi_{1},\phi_{2},\ldots ,\phi_{N}\right)\in C^{N\times N},\;\phi_{n}=e^{j\theta_{n}},\;\left|\phi_{n}\right|=1.$$

Here, $\phi_{n}=e^{j\theta_{n}}$ denotes the unit-modulus phase shift applied by the *n*-th RIS element. The unit-modulus constraint $|\phi_{n}|=1$ reflects the passive nature of the RIS, where each element changes the phase of the incident signal without active amplification. This differs from conventional active relays, which require RF chains and power amplifiers.

Since the RIS has no baseband signal processing capability, CSI acquisition is performed at the BS using the received pilot observations. The high dimensionality of the cascaded channel and the two-hop fading structure make accurate channel estimation challenging, particularly in low-signal-to-noise-ratio (SNR) regimes. This motivates the learning-based channel estimation framework considered in this work. The key notation used throughout this paper is summarized in Table 1.

### 2.2. Channel Model

Both the BS–RIS and RIS–user links are modeled as independent Rayleigh fading channels with distance-dependent large-scale path loss. The small-scale fading captures the multipath scattering of the blocked propagation environment, while the path loss accounts for the attenuation over the two physical links.

Let $d_{0}=20\;m$ denote the reference distance. The large-scale path losses of the BS–RIS link and the RIS–user link associated with user *k* are modeled as

(2) $$L_{br}={\left(\frac{d_{br}}{d_{0}}\right)}^{-\tau_{br}},\;L_{ru,k}={\left(\frac{d_{ru,k}}{d_{0}}\right)}^{-\tau_{ru}},$$

where $d_{br}$ denotes the BS–RIS distance, $d_{ru,k}$ denotes the distance between the RIS and user *k*, and $\tau_{br}$ and $\tau_{ru}$ are the corresponding path-loss exponents.

The RIS–user channel is denoted by $H_{ru}\in C^{K\times N}$, where the (k,n)-th entry $h_{ru}^{k,n}$ represents the complex baseband channel coefficient between user *k* and the *n*-th RIS element. The BS–RIS channel is denoted by $H_{br}\in C^{N\times N_{t}}$, where the (n,t)-th entry $h_{br}^{n,t}$ represents the channel coefficient between the *n*-th RIS element and the *t*-th BS antenna.

The small-scale fading coefficients are modeled as

(3) $$h_{br}^{n,t}∼\mathrm{CN}\left(0,1\right),\;h_{ru}^{k,n}∼\mathrm{CN}\left(0,1\right),$$

independently over RIS elements, BS antennas, and users.

During the uplink training phase, the pilot signal transmitted by user *k* reaches the BS through the RIS-reflected path. The signal first propagates from user *k* to the *n*-th RIS element through $h_{ru}^{k,n}$, is phase-shifted by $\phi_{n}$, and then propagates from the *n*-th RIS element to the *t*-th BS antenna through $h_{br}^{n,t}$. By summing the contributions of all RIS elements and incorporating the two-hop path loss, the effective cascaded channel between user *k* and BS antenna *t* is written as

(4) $$G_{k,t}=\sqrt{L_{br}L_{ru,k}}\sum_{n=1}^{N}h_{ru}^{k,n}\phi_{n}h_{br}^{n,t},$$

for k=1,…,K and $t=1,\ldots ,N_{t}$.

Defining

(5) $$D=\mathrm{diag}\left(\sqrt{L_{br}L_{ru,1}},\ldots ,\sqrt{L_{br}L_{ru,K}}\right),$$

the full cascaded channel matrix $G\in C^{K\times N_{t}}$ can be expressed as

(6) $$G=DH_{ru}\Phi H_{br},$$

In (6), the product $H_{ru}\Phi H_{br}$ compactly represents the RIS-reflected two-hop small-scale channel introduced above, while D incorporates the user-dependent large-scale attenuation $\sqrt{L_{br}L_{ru,k}}$. Equivalently, (6) is the compact matrix representation of (4), where each element of G is obtained by coherently summing all RIS-element reflection paths between a user and a BS antenna and then scaling the result by the corresponding two-hop path loss. This decomposition separates the distance-dependent path loss from the small-scale fading and RIS phase-shift effects, clarifying why G follows a cascaded double-fading structure rather than a conventional single-hop channel.

### 2.3. Pilot Observation and Channel Estimation Problem

Let $S\in C^{K\times T_{p}}$ denote the uplink pilot matrix transmitted by the *K* users over $T_{p}$ pilot symbols. The pilot matrix is assumed to satisfy

(7) $$SS^{H}=T_{p}I_{K},$$

which corresponds to orthogonal pilot sequences across users.

For a fixed RIS phase configuration during the pilot block, the received pilot signal at the BS is written as

(8) $$R=G^{T}S+N,$$

where $R\in C^{N_{t}\times T_{p}}$ is the received pilot signal matrix, and N denotes the additive white Gaussian noise (AWGN) matrix. The entries of N are assumed to be independent and identically distributed according to $\mathrm{CN}(0,\sigma_{z}^{2})$.

After correlating the received signal with the known pilot matrix, the BS obtains the pilot-processed noisy cascaded channel observation

(9) $$\tilde{G}={\left(\frac{1}{T_{p}}RS^{H}\right)}^{T}=G+Z,$$

where

(10) $$Z={\left(\frac{1}{T_{p}}NS^{H}\right)}^{T}$$

is the effective noise matrix after pilot processing. Therefore, the channel estimation problem considered in this work is to recover the underlying cascaded channel G from the noisy observation $\tilde{G}$.

## 3. Quantum Neural Network-Based Channel Estimation Models

### 3.1. Learning-Based Channel Estimation Framework

This work formulates cascaded channel estimation as a supervised regression problem. Given the pilot-processed noisy observation $\tilde{G}$, a learning-based estimator $f_{\Theta }\left(\cdot \right)$ predicts the cascaded channel as

(11) $$\hat{G}=f_{\Theta }\left(\tilde{G}\right),$$

where Θ denotes the trainable parameters of the estimator.

The estimator is trained by minimizing the mean-squared error between the predicted and true cascaded channels:

(12) $$\Theta^{⋆}=\arg\min_{\Theta }\frac{1}{M}\sum_{m=1}^{M}{∥G^{\left(m\right)}-f_{\Theta }\left({\tilde{G}}^{\left(m\right)}\right)∥}_{F}^{2},$$

where *M* is the number of training samples and ${∥\cdot ∥}_{F}$ denotes the Frobenius norm.

Four estimator types are considered: a QCNN, the proposed MF-QCNN, a classical one-dimensional CNN, and an MLP. The QCNN uses a single parameterized quantum convolutional circuit to extract quantum features from the channel observation. The MF-QCNN extends this structure by employing multiple independent PQC filters in parallel, allowing the model to learn complementary quantum feature representations. The CNN and MLP are considered classical neural network baselines under the same channel-estimation setting. The CNN extracts convolutional features from the reshaped input sequence, whereas the MLP estimates the cascaded channel by directly passing the normalized input vector through fully connected layers.

### 3.2. Quantum Convolutional Neural Network

**Figure 2:**
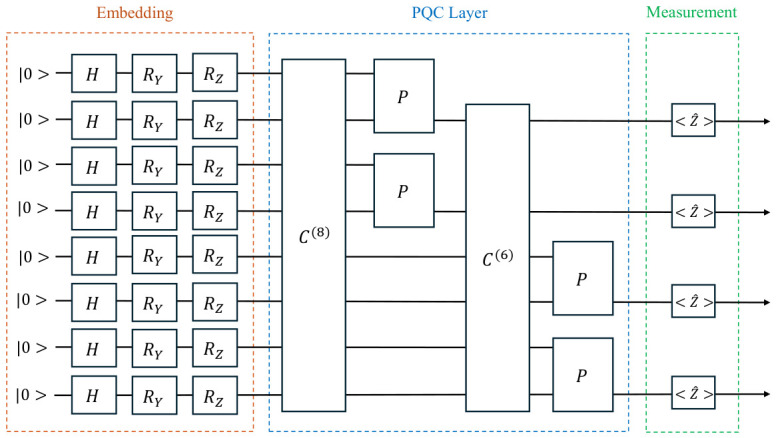
QCNN circuit architecture consisting of three stages: Embedding, PQC Layer, and Measurement.

Figure 2 illustrates the overall signal flow of the QCNN circuit used for quantum feature extraction.

In the embedding stage, the normalized real-valued channel features are assigned to eight qubits initialized as |0〉 and encoded through Hadamard, $R_{Y}$, and $R_{Z}$ gates. The encoded quantum state is then processed by the PQC layer, which consists of a first quantum convolution block $C^{\left(8\right)}$, pooling operations *P*, a second convolution block $C^{\left(6\right)}$, and additional pooling operations. Through this convolution–pooling sequence, the active-qubit set is gradually reduced from eight qubits to four representative qubits while preserving correlated channel information. Finally, the measurement stage obtains Pauli-*Z* expectation values from the remaining active qubits, and these measured quantum features are provided to the subsequent classical dense layers to estimate the cascaded channel.

#### 3.2.1. Data Preprocessing and Embedding Framework

Before entering the quantum circuit, the noisy channel observation is preprocessed into a normalized real-valued input vector, denoted by $x={\left[x_{1},\ldots ,x_{8}\right]}^{T}$. Each feature is normalized prior to encoding. A nonlinear transformation is then applied to each feature $x_{i}$ to generate two angular parameters for quantum encoding:

(13) $$\psi_{1}^{\left(i\right)}=\arctan\left(x_{i}\right),\;\psi_{2}^{\left(i\right)}=\arctan\left(x_{i}^{2}\right),$$

for i=1,…,8.

Each qubit is initialized to the state |0〉. For the *i*-th qubit, a Hadamard gate is first applied to create a superposition state, followed by $R_{Y}\left(\psi_{1}^{\left(i\right)}\right)$ and $R_{Z}\left(\psi_{2}^{\left(i\right)}\right)$ rotations for data encoding. The encoded state of the *i*-th qubit is written as

(14) $$|\phi_{i}\rangle =R_{Z}\;\left(\psi_{2}^{\left(i\right)}\right)R_{Y}\;\left(\psi_{1}^{\left(i\right)}\right)H{|0\rangle }_{i},$$

where

(15) $${H|0\rangle }_{i}=\frac{{|0\rangle }_{i}+{|1\rangle }_{i}}{\sqrt{2}}.$$

The overall embedded quantum state is expressed as

(16) $$|\Phi_{\mathrm{enc}}\left(x\right)\rangle =\overset{8}{\underset{i=1}{⨂}}|\phi_{i}\rangle .$$

This embedding scheme corresponds to angle encoding, where normalized classical channel features are mapped to quantum rotation angles before being processed by the PQC layer.

#### 3.2.2. Quantum Convolution and Entanglement

**Figure 3:**
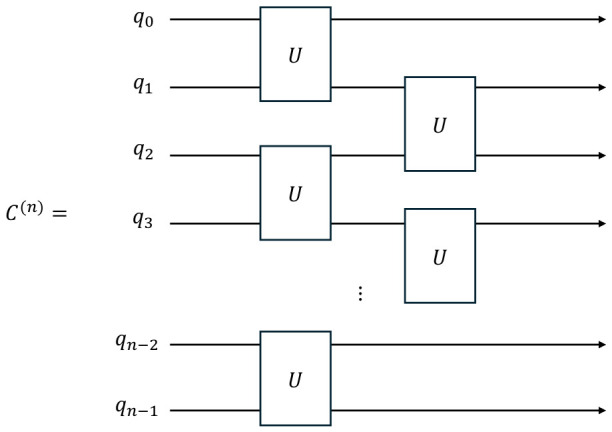
Quantum convolution layer $C^{\left(n\right)}$, where a shared parameterized two-qubit unitary block *U* is applied to neighboring active qubit pairs.

After data embedding, the encoded state is processed by the PQC layer. The quantum convolution layer $C^{\left(n\right)}$, depicted in Figure 3, applies a shared trainable two-qubit unitary block *U* to neighboring qubit pairs among the active qubits.

As shown in Figure 3, the same unitary block *U* is repeatedly applied across the active qubit sequence. The first pass connects non-overlapping neighboring pairs, such as $\left(q_{0},q_{1}\right)$ and $\left(q_{2},q_{3}\right)$, together with the remaining non-overlapping adjacent pairs in the active sequence. The second pass shifts the pairing pattern by one qubit, allowing neighboring pairs that were not connected in the first pass to interact. Finally, the boundary pair connecting the last and first active qubits is also processed so that information can circulate across the entire active-qubit sequence within one convolution layer.

**Figure 4:**
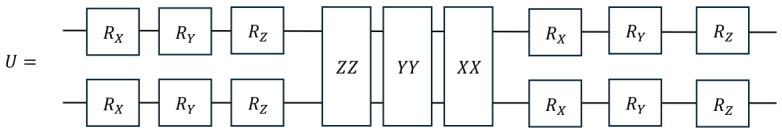
Two-qubit unitary block *U* consisting of single-qubit rotations and Ising-type entangling gates.

The internal structure of the two-qubit unitary block *U* is illustrated in Figure 4. The unitary block consists of two layers of single-qubit rotations and three Ising-type entangling gates. In the first rotation layer, each qubit is transformed by successive $R_{X}$, $R_{Y}$, and $R_{Z}$ rotations. The two qubits are then coupled through ZZ, YY, and XX entangling gates. After the entangling gates, a second layer of $R_{X}$, $R_{Y}$, and $R_{Z}$ rotations is applied to both qubits.

The entangling gates allow the circuit to model interactions between neighboring channel features encoded on different qubits. Within each convolution layer, the same parameterized unitary structure is shared across all pairwise applications, following a parameter-sharing operation similar to kernel sharing in classical CNNs. Different convolution layers use independent trainable parameter sets.

#### 3.2.3. Quantum Pooling and Measurement

**Figure 5:**
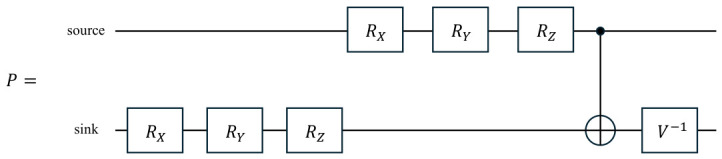
Quantum pooling layer *P* with source and sink qubits, source-to-sink CNOT gate, and inverse sink-basis transformation $V^{-1}$.

The quantum pooling layer *P*, shown in Figure 5, reduces the number of active qubits by transferring information from a source qubit to a sink qubit. For each source–sink pair, trainable single-qubit basis transformations are first applied to both the source and sink qubits. A controlled-NOT (CNOT) gate is then applied with the source qubit as the control and the sink qubit as the target, allowing the sink qubit to capture information correlated with the source qubit. Finally, the inverse of the sink-basis transformation, denoted by $V^{-1}$, is applied only to the sink qubit. Here, $V^{-1}$ denotes the inverse of the trainable basis selector on the sink qubit, not the inverse of the entire two-qubit pooling operation. In the implemented pooling block, the source and sink basis transformations are parameterized by successive $R_{X}$, $R_{Y}$, and $R_{Z}$ rotations.

After each pooling operation, the source qubit is treated as inactive: it remains in the quantum register but is excluded from subsequent convolution layers and from the final measurement set. The sink qubits therefore carry the compressed representation used by the following layers. This progressive reduction of active qubits compresses the quantum feature representation while preserving relevant information in the remaining sink qubits.

In this work, the complete QCNN processes 8 qubits through two convolution-pooling stages, followed by measurement:

- Stage 1: $C^{\left(8\right)}$ is applied to all 8 qubits. Then, two pooling operations, $q_{0}\to q_{1}$ and $q_{2}\to q_{3}$, are performed. The active qubit set after this stage is $\left\{q_{1},q_{3},q_{4},q_{5},q_{6},q_{7}\right\}$.
- Stage 2: $C^{\left(6\right)}$ is applied to the 6 active qubits. Then, two additional pooling operations, $q_{4}\to q_{5}$ and $q_{6}\to q_{7}$, are performed. The active qubit set after this stage is $\left\{q_{1},q_{3},q_{5},q_{7}\right\}$.
- Stage 3: Pauli-*Z* expectation values are measured on the four remaining active qubits, producing the quantum feature vector
  $$q={\left[\langle {\hat{Z}}_{q_{1}}\rangle ,\;\langle {\hat{Z}}_{q_{3}}\rangle ,\;\langle {\hat{Z}}_{q_{5}}\rangle ,\;\langle {\hat{Z}}_{q_{7}}\rangle \right]}^{T}.$$

For the considered two-user, two-BS-antenna configuration, the real and imaginary components of the cascaded channel form $2KN_{t}=8$ real-valued entries. Accordingly, the measured quantum feature vector is decoded by two fully connected layers, Dense(32) and Dense(16), with rectified linear unit (ReLU) activation functions. A final linear Dense(8) output layer produces the real-valued estimate of the cascaded channel, corresponding to the real and imaginary components of the 2×2 channel matrix $\hat{G}$.

The data-embedding rotations are determined by the input features, whereas the convolution and pooling rotations are trainable. The quantum and classical trainable parameters are optimized end-to-end, with gradients for quantum parameters computed using the parameter-shift rule.

### 3.3. Multi-Filter Quantum Convolutional Neural Network

**Figure 6:**
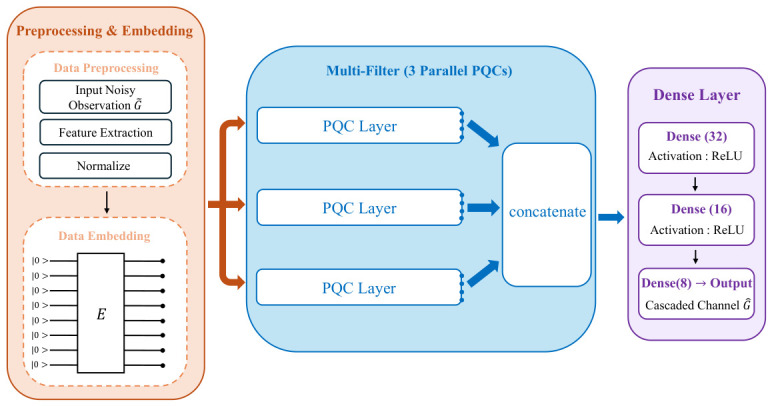
Architecture of the proposed MF-QCNN for F=3 parallel PQC filters with shared embedding and classical dense head.

To obtain a performance benefit beyond what a single PQC filter can provide, this paper proposes the MF-QCNN. Rather than deepening the circuit, which would increase decoherence sensitivity in near-term quantum devices, the MF-QCNN employs *F* independent PQC filters operating in parallel on the same embedded quantum input, as illustrated in Figure 6 for F=3. The number of filters *F* is treated as a configurable architectural hyperparameter.

The preprocessing and embedding stage is shared across all filters so that each filter receives the same encoded state $|\Phi_{\mathrm{enc}}\left(x\right)\rangle$ as input. Each filter f∈{1,…,F} has its own independently trainable parameter set $\theta^{\left(f\right)}$ and executes the same two-stage convolution-pooling structure as the single-filter QCNN, producing a 4-dimensional quantum feature vector

(17) $$q^{\left(f\right)}={\left[{\langle {\hat{Z}}_{q_{1}}\rangle }^{\left(f\right)},\;{\langle {\hat{Z}}_{q_{3}}\rangle }^{\left(f\right)},\;{\langle {\hat{Z}}_{q_{5}}\rangle }^{\left(f\right)},\;{\langle {\hat{Z}}_{q_{7}}\rangle }^{\left(f\right)}\right]}^{T}\in R^{4}.$$

The outputs of all filters are concatenated into a 4F-dimensional joint representation

(18) $$q_{\mathrm{cat}}={\left[q^{(1)T},\;\ldots ,\;q^{(F)T}\right]}^{T}\in R^{4F}.$$

The concatenated representation is decoded by a shared classical dense head consisting of Dense(32), Dense(16), and a final linear output layer of dimension 8. The dense-head layer widths are kept identical for all filter configurations. However, because the concatenated quantum feature dimension increases from 4 to 4F, the input dimension of the first dense layer scales with *F*.

For the implemented F=3 configuration, each PQC filter produces four Pauli-*Z* expectation values. Thus, the concatenated quantum feature vector has dimension 4F=12. The classical dense head then maps $q_{\mathrm{cat}}\in R^{12}$ to $h_{1}\in R^{32}$ and $h_{2}\in R^{16}$, followed by the final linear output vector $\hat{g}\in R^{8}$. The entries of $\hat{g}$ correspond to the real and imaginary components of the estimated cascaded channel coefficients over all user–antenna pairs. Finally, $\hat{g}$ is reshaped into $\hat{G}\in C^{K\times N_{t}}$. This filter-wise expansion differs from increasing the depth of a single PQC because the additional filters are evaluated in parallel, and each filter has an independent parameter set. Consequently, the feature dimension increases from 4 to 4F, while the depth of each individual PQC filter remains unchanged.

The complete inference procedure is summarized in Algorithm 1. Starting from the noisy observation $\tilde{G}$, real and imaginary features are extracted and normalized to form x (Step 1). The normalized features are mapped to encoding angles via arctan transformations and used to prepare the shared embedded quantum state $|\Phi_{\mathrm{enc}}\rangle$ (Step 2). Each of the *F* parallel PQC filters independently processes $|\Phi_{\mathrm{enc}}\rangle$ through two convolution-pooling stages and measures the Pauli-*Z* expectation values on the four remaining active qubits to yield $q^{\left(f\right)}$ (Step 3). The filter outputs are concatenated into $q_{\mathrm{cat}}$ (Step 4) and passed through the classical dense head to produce $\hat{G}$ (Step 5).

For notational compactness, the pooling parameters in Algorithm 1 are grouped by pooling stage. In the implemented MF-QCNN architecture, each source–sink pooling operation uses its own independently trainable parameter set, and no parameter sharing is intended among different pooling operations. Also, the notation $\langle Z_{i}\rangle$ in the algorithm denotes the Pauli-*Z* expectation value measured on the corresponding active qubit $q_{i}$. The MF-QCNN is trained end-to-end by jointly optimizing all quantum filter parameters ${\left\{\theta^{\left(f\right)}\right\}}_{f=1}^{F}$ and the classical dense head parameters. For each trainable quantum parameter in a PQC filter, the parameter-shift rule computes the derivative of the measured PQC outputs from two circuit evaluations obtained by shifting only that parameter by +π/2 and −π/2, while keeping all other parameters fixed. Thus, each quantum parameter requires two shifted PQC evaluations, whereas gradients for the classical dense-head parameters are obtained through standard backpropagation. This parallel-filter structure provides a configurable way to adjust the quantum feature dimension according to the available quantum resources.
**Algorithm 1** Multi-filter quantum convolutional neural network for cascaded channel estimation**Input:** $\tilde{G}$: pilot-processed noisy cascaded channel observation; *F*; ${\left\{\theta^{\left(f\right)}\right\}}_{f=1}^{F}$, $\Theta_{\mathrm{dense}}$**Output:** $\hat{G}$: estimated cascaded channel matrix **Step** **1:****Preprocessing**  1:Extract real and imaginary parts from $\tilde{G}$ by stacking over all (k,t) pairs:x=Re(G˜k,t),Im(G˜k,t)k=1,…,K;t=1,…,Nt  2:Normalize the feature vector x **Step** **2:****Data Embedding (shared across all filters)**  3:Compute encoding angles: $\psi_{1}^{\left(i\right)}\leftarrow \arctan\left(x_{i}\right)$,   $\psi_{2}^{\left(i\right)}\leftarrow \arctan\left(x_{i}^{2}\right)$,   i=1,…,8  4:Prepare embedded quantum state via angle encoding:$$|\Phi_{\mathrm{enc}}\rangle \leftarrow \overset{8}{\underset{i=1}{⨂}}R_{Z}\;\left(\psi_{2}^{\left(i\right)}\right)R_{Y}\;\left(\psi_{1}^{\left(i\right)}\right)H\;{|0\rangle }_{i}$$ **Step** **3:****PQC Layer—Convolution and Pooling**  5:**for** f=1 **to** *F* **do**  6:    *// Convolution Stage 1*  7:    Apply $C^{\left(8\right)}$ with shared unitary $U(\theta_{c1}^{\left(f\right)})$ to all 8 qubits  8:    *// Pooling Stage 1*  9:    Apply *P*:$q_{0}\;\to \;q_{1}$,$q_{2}\;\to \;q_{3}$ with $V(\theta_{p1}^{\left(f\right)})$ (active: $\left\{q_{1},q_{3},q_{4},q_{5},q_{6},q_{7}\right\}$)10:    *// Convolution Stage 2*11:    Apply $C^{\left(6\right)}$ with shared unitary $U(\theta_{c2}^{\left(f\right)})$ to 6 active qubits12:    *// Pooling Stage 2*13:    Apply *P*:$q_{4}\;\to \;q_{5}$,$q_{6}\;\to \;q_{7}$ with $V(\theta_{p2}^{\left(f\right)})$ (active: $\left\{q_{1},q_{3},q_{5},q_{7}\right\}$)14:**end for****Step** **4:****Measurement and Concatenation**15:**for** f=1 **to** *F* **do**16:    Measure Pauli-*Z* on $\left\{q_{1},q_{3},q_{5},q_{7}\right\}$: $q^{\left(f\right)}\leftarrow {\left[{\langle Z_{1}\rangle }^{\left(f\right)},\;{\langle Z_{3}\rangle }^{\left(f\right)},\;{\langle Z_{5}\rangle }^{\left(f\right)},\;{\langle Z_{7}\rangle }^{\left(f\right)}\right]}^{T}$17:**end for**18:Concatenate filter outputs: $q_{\mathrm{cat}}\leftarrow {\left[q^{(1)T},\;\ldots ,\;q^{(F)T}\right]}^{T}$**Step** **5:****Classical Dense Decoding**19:$h_{1}\leftarrow \mathrm{ReLU}\left(W_{1}\;q_{\mathrm{cat}}+b_{1}\right)$    (Dense(32), activation: ReLU)20:$h_{2}\leftarrow \mathrm{ReLU}\left(W_{2}\;h_{1}+b_{2}\right)$    (Dense(16), activation: ReLU)21:$\hat{g}\leftarrow W_{\mathrm{out}}\;h_{2}+b_{\mathrm{out}}$    (Dense(8), activation: Linear)22:$\hat{G}\leftarrow \mathrm{reshape}\left(\hat{g}\right)$23:**return** $\hat{G}$

### 3.4. Classical CNN and MLP Baselines

**Figure 7:**
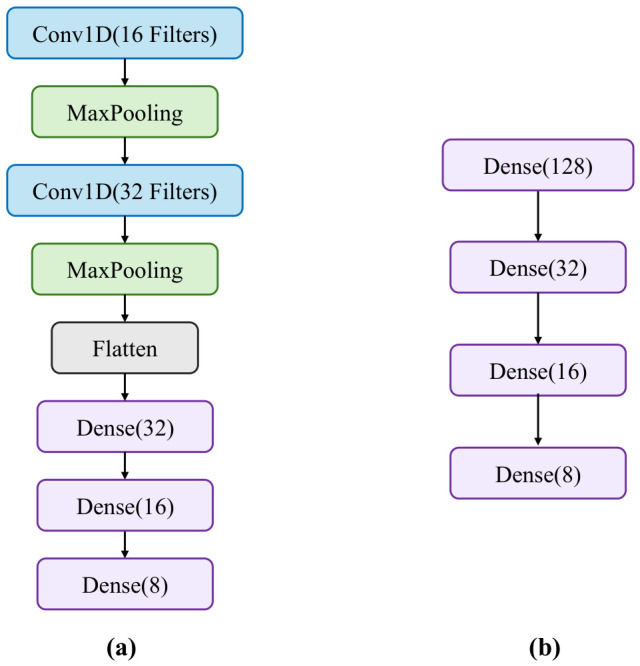
Architectures of the classical baselines: (**a**) CNN and (**b**) MLP.

Two classical neural-network baselines are considered under the same cascaded channel estimation setting: a one-dimensional CNN and an MLP. Both models operate entirely in the classical domain and share the same input preprocessing and output representation as the quantum-based estimators. The architectures of the CNN and MLP baselines are illustrated in Figure 7.

For the CNN baseline, the preprocessed and normalized input vector $x\in R^{8}$ is reshaped into a length-8 sequence with a single feature channel before being fed into the network. The feature extraction stage consists of two convolutional blocks, each comprising a one-dimensional convolutional layer followed by max-pooling. The first block applies 16 filters with a kernel size of 3 and same-padding, followed by max-pooling with pool size 3 and stride 1. The second block applies 32 filters with the same kernel configuration, again followed by max-pooling with a pool size of 3 and a stride of 1. Both convolutional layers use ReLU activation functions.

The pooled feature maps are subsequently flattened into a one-dimensional vector and passed through two fully connected layers, Dense(32) and Dense(16), both with ReLU activations. A final linear output layer produces $2KN_{t}$ real-valued entries, corresponding to the real and imaginary components of $\hat{G}\in C^{K\times N_{t}}$.

For the MLP baseline, the same normalized input vector $x\in R^{8}$ is directly processed by fully connected layers without convolution or pooling operations. The MLP consists of Dense(128), Dense(32), Dense(16), and a final linear Dense(8) output layer. The hidden layers use ReLU activation functions, and the final output layer produces the real-valued estimate of the cascaded channel. For the considered K=2, $N_{t}=2$ configuration, the output dimension of both classical baselines is 8.

Both classical baselines share the same dense decoding head and output representation but differ in their feature-extraction mechanisms: convolutional feature extraction for the CNN and fully connected feature transformation for the MLP. They are trained end-to-end by minimizing the mean squared error between the predicted and true cascaded channels. Unlike the QCNN and MF-QCNN, which rely on PQCs for feature extraction, the CNN and MLP perform feature extraction entirely through classical operations, with all gradients computed via standard backpropagation.

## 4. Simulation Setup

The simulation environment is configured to evaluate cascaded channel estimation performance in a RIS-assisted multi-user uplink system. All simulations were implemented in Python 3.10 using TensorFlow 2.15.0 and TensorFlow Quantum. The BS is equipped with $N_{t}=2$ antennas and serves K=2 single-antenna users. The RIS comprises N=32 passive reflecting elements. The reference distance is set to $d_{0}=20\;m$, the BS–RIS distance to $d_{br}=50\;m$, and the RIS–user distances to $d_{ru,1}=25\;m$ and $d_{ru,2}=30\;m$. The BS–RIS and RIS–user path-loss exponents are set to $\tau_{br}=2.2$ and $\tau_{ru}=2.8$, respectively. Both links are modeled as independent Rayleigh fading channels with unit variance, i.e., $h_{br}^{n,t}∼\mathrm{CN}\left(0,1\right)$ and $h_{ru}^{k,n}∼\mathrm{CN}\left(0,1\right)$.

A cascaded channel dataset of 50,000 samples is generated. The dataset is divided into 35,000 training samples, 7500 validation samples, and 7500 test samples, corresponding to a 70%/15%/15% split. For the learning-based channel-estimation techniques, noisy cascaded channel observations are used as inputs, while the corresponding noiseless cascaded channels are used as labels. After training, the techniques are evaluated across SNR values ranging from −10dB to 30dB in steps of 5dB.

The primary channel reconstruction metric is the user- and antenna-averaged NMSE, defined as

(19) $$\mathrm{NMSE}=\frac{1}{KN_{t}}\sum_{k=1}^{K}\sum_{t=1}^{N_{t}}\frac{E\left[{\left|G_{k,t}-{\hat{G}}_{k,t}\right|}^{2}\right]}{E\left[{\left|G_{k,t}\right|}^{2}\right]},$$

where $G_{k,t}$ and ${\hat{G}}_{k,t}$ denote the true and reconstructed cascaded channel coefficients for user *k* and BS antenna *t*, respectively. The expectation E[·] denotes averaging over the evaluation channel and noise conditions. For plotting and comparison, the NMSE is reported in dB as

(20) $${\mathrm{NMSE}}_{\mathrm{dB}}=10\log_{10}\left(\mathrm{NMSE}\right).$$

All NMSE results are obtained by first converting the per-trial NMSE to the dB scale and then computing the sample mean and sample standard deviation over 1000 Monte Carlo trials in the dB domain. Error bars indicate ±1 standard deviation.

All learning-based channel-estimation techniques are trained under the same training protocol for fair comparison. Specifically, the CNN, MLP, QCNN, and MF-QCNN models are trained for 100 epochs using the Adam optimizer with a learning rate of $\eta =2\times 10^{-3}$ and the same batch size. No early stopping is applied, and the learning rate, batch size, and number of epochs are fixed globally rather than tuned separately for each model. For the QCNN and MF-QCNN, quantum parameter gradients are computed via the parameter-shift rule, while classical parameter gradients are obtained through standard backpropagation. For the filter-sweep comparison, the single-filter QCNN is regarded as the F=1 case, while the proposed MF-QCNN is evaluated with F∈{2,3,4} parallel PQC filters. The simulation parameters in Table 2 are selected as the key evaluation basis for RIS-assisted cascaded channel estimation. The numbers of users *K*, BS antennas $N_{t}$, and RIS reflecting elements *N* determine the cascaded-channel dimension, while the link distances and path-loss exponents define the large-scale attenuation of the RIS-assisted two-hop channel. The SNR range and Monte Carlo trials are used to evaluate reconstruction accuracy under different noise levels with statistical reliability. Finally, the number of qubits and filter-sweep configurations specify the quantum input dimension and the resource–accuracy trade-off of the proposed MF-QCNN. Thus, Table 2 provides the basis for interpreting the NMSE, achievable sum rate, complexity, and quantum-resource scaling results under consistent evaluation conditions.

## 5. Results

### 5.1. Training Convergence

**Figure 8:**
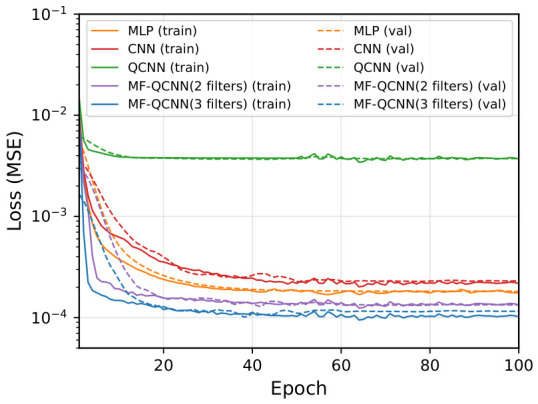
Training and validation loss curves for the representative learning-based channel-estimation techniques over 100 epochs.

Figure 8 illustrates the training and validation loss curves of the representative learning-based channel-estimation techniques over 100 epochs.

As shown in Figure 8, all techniques exhibit rapid initial convergence followed by gradual stabilization, while the CNN converges more gradually than the other models over the first 40–50 epochs. The training and validation losses remain closely aligned for each technique throughout the 100 epochs, indicating that severe overfitting is not observed.

The single-filter QCNN reaches a stable plateau within the first 5–10 epochs but remains at the highest loss level among the compared techniques. This result suggests that a single shallow PQC filter has limited capacity to represent the cascaded double-fading channel structure. The CNN achieves a lower loss than the single-filter QCNN, and the MLP also maintains a lower loss level than the CNN in this experiment.

Including both CNN and MLP baselines allows the proposed MF-QCNN to be compared with different classical neural-network architectures under the same vectorized input representation. These baselines are intentionally evaluated in their uncompressed forms, without pruning, quantization, or depthwise separable convolution. Nevertheless, they provide meaningful reference points for parameter efficiency because CNN- and MLP-based estimators are representative learning-based architectures that have been widely used as classical baselines in wireless channel-estimation studies, including RIS-assisted channel-estimation scenarios. Therefore, the comparison clarifies how the proposed MF-QCNN performs against standard classical neural-network estimators before introducing additional compression-specific design choices.

The MF-QCNN with 2 filters further lowers both training and validation loss compared with the classical baselines, suggesting that the parallel PQC-filter structure contributes to the learned feature representation. The MF-QCNN with 3 filters achieves the lowest loss among the curves shown. However, the gap between the 2-filter and 3-filter MF-QCNN curves is relatively small compared with the gap between the single-filter QCNN and the multi-filter configurations. This trend suggests that increasing the number of PQC filters improves convergence, while the additional gain becomes more moderate as the filter count increases. The slower convergence and higher final loss of the CNN suggest that local one-dimensional kernels are less expressive than the parallel quantum filters for this eight-dimensional vectorized input.

### 5.2. NMSE Performance

**Figure 9:**
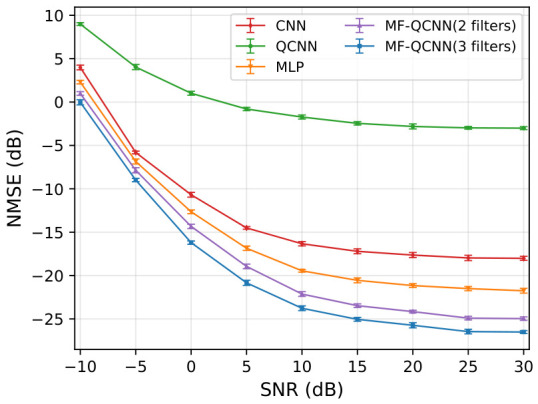
NMSE comparison versus SNR for the main learning-based channel-estimation techniques.

Figure 9 compares the NMSE performance of the main learning-based channel-estimation techniques as a function of SNR.

Since the NMSE definition in (19) and its dB conversion in (20) are provided in Section 4, this subsection focuses on the relative reconstruction accuracy among the compared techniques. Among the techniques shown in Figure 9, the MF-QCNN with 3 filters achieves the lowest NMSE across the entire SNR range, followed by the MF-QCNN with 2 filters. This trend indicates that using multiple parallel PQC filters improves the quality of the learned quantum feature representation.

The single-filter QCNN shows the weakest NMSE performance among the learning-based techniques in Figure 9. Although its NMSE decreases at low SNR, it quickly reaches a high-SNR error floor of approximately −2.8dB. This saturation suggests that a single PQC filter alone is insufficient to accurately reconstruct the cascaded channel when the noise level becomes small. Both classical baselines continue to improve as the SNR increases. At SNR=20dB, the CNN baseline reaches approximately −17.6dB, while the MLP baseline achieves approximately −21.1dB. Thus, the MLP shows lower NMSE than the CNN for the considered vectorized channel input, but it still remains less accurate than the MF-QCNN configurations.

The MF-QCNN techniques provide a clear improvement over the single-filter QCNN and the classical baselines. At SNR=20dB, the MF-QCNN with 3 filters achieves an NMSE of approximately −25.7dB, which corresponds to improvements of about 22.9dB, 8.1dB, and 4.6dB over the single-filter QCNN, CNN, and MLP baselines, respectively. At this SNR point, the corresponding dB-domain standard deviations of the compared NMSE curves range from approximately 0.15–0.30dB. These results show that the proposed multi-filter structure effectively mitigates the representational limitation of the single-filter QCNN and improves cascaded channel reconstruction accuracy across both low- and high-SNR regimes.

### 5.3. Effect of the Number of Filters

**Figure 10:**
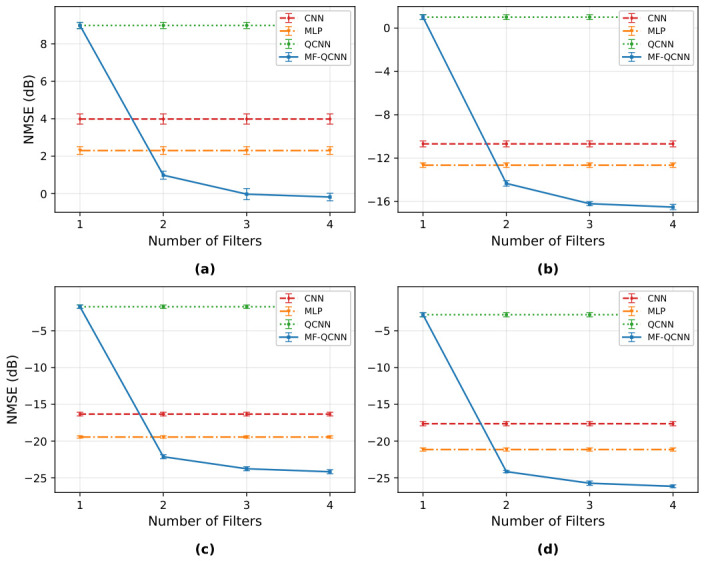
NMSE (dB) versus the number of PQC filters for CNN, MLP, QCNN, and MF-QCNN at (**a**) SNR = −10 dB, (**b**) SNR = 0 dB, (**c**) SNR = 10 dB, and (**d**) SNR = 20 dB.

Figure 10 examines the effect of the number of PQC filters on the NMSE performance of the compared channel-estimation techniques.

In this comparison, the single-filter QCNN is treated as the F=1 case, while the MF-QCNN corresponds to configurations with multiple parallel PQC filters. For the CNN, MLP, and single-filter QCNN, the same performance values are shown across the filter axis because their architectures do not vary with the MF-QCNN filter count. The filter sweep is conducted for F∈{1,2,3,4} at four selected SNR values, as shown in Figure 10a–d.

The largest improvement occurs when the number of filters increases from F=1 to F=2 across all SNR conditions. For example, at SNR=20dB, the NMSE decreases from approximately −2.8dB for the single-filter QCNN to around −24.2dB for the 2-filter MF-QCNN, which already surpasses the CNN and MLP baselines. This result shows that adding a single parallel PQC filter significantly improves the reconstruction accuracy.

Further increasing the filter count from F=2 to F=3 continues to reduce the NMSE. At SNR=20dB, the 3-filter MF-QCNN achieves an NMSE of approximately −25.7dB, corresponding to about 1.6dB improvement over the 2-filter configuration. When the filter count is increased from F=3 to F=4, the NMSE decreases further, but the improvement becomes relatively small compared with the gain observed in the transition from F=1 to F=2. This indicates a clear diminishing-return behavior as the number of PQC filters increases.

The separation between different filter configurations is relatively small at low SNR, as shown in Figure 10a, whereas the performance gap becomes more pronounced in the moderate- and high-SNR regimes shown in Figure 10c–d. Overall, Figure 10 shows that increasing the number of parallel PQC filters consistently improves cascaded channel reconstruction accuracy beyond the CNN, MLP, and QCNN baselines, but the performance gain becomes progressively smaller beyond the 3-filter configuration.

### 5.4. Complexity Analysis

The complexity of the MLP, CNN, QCNN, and MF-QCNN models is assessed from two complementary viewpoints: the number of trainable parameters, which reflects model size, and the per-sample computational cost, which reflects runtime effort.

For the CNN, the total number of trainable parameters is determined by two Conv1D layers and three dense layers. The first convolutional layer applies 16 filters of kernel size 3 to a single-channel input, contributing 3·1·16+16=64 parameters, and the second convolutional layer applies 32 filters to the 16-channel output, contributing 3·16·32+32=1568 parameters. After two max-pooling operations, the feature map is flattened to a 128-dimensional vector, and the three dense layers contribute 128·32+32=4128, 32·16+16=528, and 16·8+8=136 parameters, respectively. The total parameter count for the CNN is therefore 64+1568+4128+528+136=6424.

For the MLP baseline, the input vector has dimension 8 and is processed by Dense(128), Dense(32), Dense(16), and Dense(8) layers. The corresponding trainable-parameter counts are 8·128+128=1152, 128·32+32=4128, 32·16+16=528, and 16·8+8=136, respectively. Therefore, the total number of trainable parameters in the MLP is 1152+4128+528+136=5944.

For the QCNN, the classical convolutional feature extractor is replaced by an 8-qubit PQC followed by the same dense decoder. The quantum circuit contains 54 trainable parameters distributed across two convolutional layers and four pooling operations. Since the circuit outputs a 4-dimensional measurement vector, the three dense layers contribute 4·32+32=160, 32·16+16=528, and 16·8+8=136 parameters, respectively, giving a dense total of 160+528+136=824. The overall QCNN parameter count is therefore 54+824=878, representing an 86.3% reduction relative to the CNN.

For the MF-QCNN with *F* parallel quantum filters, the quantum parameters scale linearly with *F*, contributing 54F parameters in total. The first dense layer receives a 4F-dimensional concatenated input and contributes 4F·32+32=128F+32 parameters, while the remaining two dense layers contribute a fixed 528+136=664 parameters. The overall parameter count is therefore 54F+(128F+32)+664=182F+696, giving 1060, 1242, and 1424 trainable parameters for F=2, F=3, and F=4, respectively.

**Figure 11:**
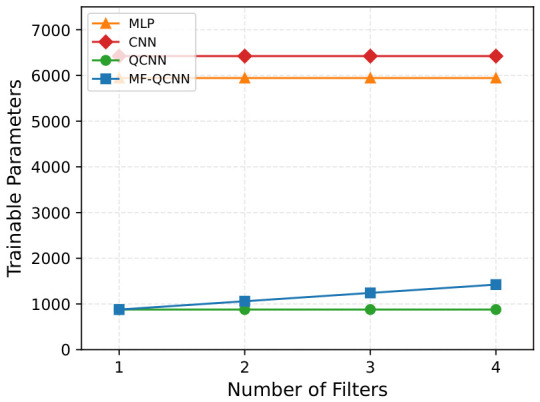
Trainable parameter count versus the number of filters for the MLP, CNN, QCNN, and MF-QCNN models.

Figure 11 illustrates the trainable parameter counts of the MLP, CNN, QCNN, and MF-QCNN models as a function of the number of filters. The MLP and CNN have fixed counts of 5944 and 6424 parameters, respectively, regardless of the MF-QCNN filter count, while the QCNN has 878 parameters. The MF-QCNN grows linearly with *F* but stays well below both classical baselines across all tested filter configurations.

A small trainable-parameter count, however, does not by itself imply a low computational cost. To clarify this distinction, the per-sample computational cost is also examined in terms of circuit evaluations and floating-point operations (FLOPs).

For the quantum models, the dominant cost is the number of PQC circuit evaluations. During inference, each MF-QCNN sample requires one forward circuit evaluation per parallel filter, so the number of circuit evaluations grows linearly as *F*, i.e., 1, 2, and 3 evaluations per sample for F=1, 2, and 3, respectively. During training, the gradient of every quantum-circuit parameter is obtained with the parameter-shift rule, which evaluates each parameter at two shifted points and therefore requires two additional circuit evaluations per parameter. Since each filter carries 54 trainable quantum parameters, a single training step entails approximately F+2·54F circuit evaluations per sample, i.e., about 109, 218, and 327 evaluations for F=1, 2, and 3. This cost grows linearly with *F*, but carries a large multiplicative constant that has no counterpart in classical backpropagation, where the MLP and CNN instead require only a single forward and backward pass and therefore involve no circuit evaluations.

To place the quantum and classical models on a common scale, we estimate the cost of a single 8-qubit circuit evaluation under dense statevector simulation, which is the setting used in this work. In this model, the quantum state is stored as a vector of $2^{8}=256$ complex amplitudes, and each gate is applied as a sparse matrix–vector update on these amplitudes. A single-qubit gate updates the amplitudes in $2^{7}$ pairs through a 2×2 complex multiplication, which amounts to roughly 3.6 kFLOP, whereas a two-qubit gate acts on $2^{6}$ amplitude groups through a 4×4 complex multiplication, amounting to roughly 7.7 kFLOP, where one complex multiplication is counted as six real FLOPs and one complex addition as two real FLOPs. Counting the angle-encoding embedding, convolution, and pooling gates in one PQC filter (228 single-qubit and 46 two-qubit gates) yields approximately 1.17 MFLOP per circuit evaluation. Consequently, the simulated inference cost of the 3-filter MF-QCNN is on the order of 3.51 MFLOP per sample, compared with about 11.5 kFLOP for the MLP and 28.7 kFLOP for the CNN.

The trainable-parameter counts and per-sample computational costs of all models are summarized together in Table 3. As shown in the table, although the 3-filter MF-QCNN uses only about 19.3% of the CNN trainable parameters, its simulated inference cost is roughly two orders of magnitude higher, because each quantum-circuit evaluation involves preparing and propagating a full quantum state rather than a single classical forward pass. The compact parameter count of the MF-QCNN therefore reflects model size rather than runtime cost. Moreover, the reported FLOP figures correspond to the cost of classically simulating the quantum circuits: a dense statevector simulator stores all $2^{n}$ amplitudes explicitly, so its cost grows exponentially with the number of qubits. On physical quantum hardware, the same *n*-qubit state is represented by *n* qubits rather than $2^{n}$ classical amplitudes, and the relevant cost is instead measured in gate operations and measurement shots, which have no direct FLOP equivalent. The FLOP comparison should thus be read as an order-of-magnitude indication of present-day simulation cost, not as a statement about the intrinsic cost of executing the MF-QCNN on quantum hardware.

### 5.5. Achievable Sum Rate

In addition to NMSE, the achievable sum rate is evaluated to examine the effect of the reconstructed cascaded channel on downlink transmission performance. For each channel-estimation technique, the BS designs a ZF precoder using the estimated cascaded channel $\hat{G}$, while the received signal quality is evaluated through the true cascaded channel G. Given $\hat{G}\in C^{K\times N_{t}}$, the ZF precoding matrix is obtained as

(21) $$\tilde{W}={\hat{G}}^{H}{\left(\hat{G}{\hat{G}}^{H}\right)}^{-1}=\left[{\tilde{w}}_{1},\ldots ,{\tilde{w}}_{K}\right],$$

where ${\tilde{w}}_{k}$ denotes the unnormalized precoding vector for user *k*. Each precoding vector is then normalized as

(22) $$w_{k}=\frac{{\tilde{w}}_{k}}{{∥{\tilde{w}}_{k}∥}_{2}},\;k=1,\ldots ,K.$$

Let $g_{k}^{T}$ be the *k*-th row of the true cascaded channel G. With equal power allocation and normalized noise variance, the downlink signal-to-interference-plus-noise ratio (SINR) of user *k* is given by

(23) $${\mathrm{SINR}}_{k}=\frac{\frac{P}{K}{\left|g_{k}^{T}w_{k}\right|}^{2}}{\frac{P}{K}\sum_{j\neq k}{\left|g_{k}^{T}w_{j}\right|}^{2}+1},$$

where *P* denotes the total downlink transmit power. The achievable sum rate is calculated as

(24) $$R_{\mathrm{sum}}=\sum_{k=1}^{K}\log_{2}\left(1+{\mathrm{SINR}}_{k}\right).$$

**Figure 12:**
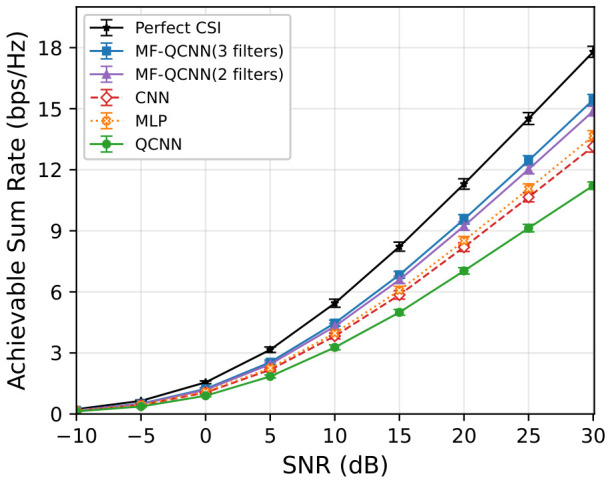
Achievable sum rate versus SNR under downlink ZF precoding.

Figure 12 shows the achievable sum rate versus SNR under downlink ZF precoding. The perfect CSI curve represents the reference case where the true cascaded channel is used for precoder design. Among the compared techniques, MF-QCNN with 3 filters achieves the highest sum rate over the entire SNR range. This result shows that the improved channel reconstruction accuracy of the proposed MF-QCNN leads to higher downlink spectral efficiency.

At low SNR, the performance gap between the techniques is small because the received signal is mainly limited by noise. At SNR=30dB, the CNN and MLP baselines achieve approximately 13.14bps/Hz and 13.68bps/Hz, respectively, while MF-QCNN with 2 filters and MF-QCNN with 3 filters achieve approximately 14.88bps/Hz and 15.43bps/Hz, respectively. Compared with the CNN baseline, MF-QCNN with 2 filters and MF-QCNN with 3 filters increase the achievable sum rate by approximately 13.2% and 17.4%, respectively. The 3-filter MF-QCNN also improves the achievable sum rate by approximately 12.8% over the MLP baseline.

At SNR=30dB, the standard deviations of the achievable sum rate for these methods are approximately 0.23–0.28bps/Hz. Therefore, the proposed multi-filter quantum technique improves not only the NMSE performance but also the achievable downlink sum rate.

This gain is consistent with the ZF precoder’s reliance on the estimated channel $\hat{G}$ in (21), while the SINR in (23) is evaluated with the true channel G. When the channel estimate is more accurate, the resulting precoder is more closely matched to the true cascaded channel. Accordingly, the lower NMSE of the 3-filter MF-QCNN is reflected in its higher achievable sum rate.

## 6. Discussion

The proposed MF-QCNN is designed with near-term quantum-resource constraints in mind, rather than assuming access to large, fault-tolerant quantum processors. Each PQC filter uses only eight qubits and a fixed, shallow convolution-pooling structure, so the circuit depth of each filter does not increase with the filter count *F*, and the trainable-parameter count scales linearly as 182F+696 for the considered system configuration. This design is intended to be more conservative than simply deepening a single PQC, given the gate-noise and barren-plateau issues commonly associated with deeper NISQ circuits [39,40,41].

As discussed in the introduction, the novelty of MF-QCNN lies in combining multiple independently trainable shallow PQC filters in parallel, rather than deepening a single circuit or stacking quantum and classical stages serially, and in applying this structure to continuous-valued cascaded channel estimation in an RIS-assisted multi-user system rather than to image classification. The NMSE and sum-rate results are reported with error bars representing ±1 sample standard deviation over the evaluation trials. Together with the parameter-efficiency results reported in Section 5, they indicate that this design choice provides a favorable accuracy–resource trade-off in the considered simulation setting.

To further position the proposed approach with respect to classical pruning-based compression, we note that reducing the CNN baseline to the same trainable-parameter level as the 3-filter MF-QCNN would require the CNN to retain only 1242/6424≈19.3% of its parameters, corresponding to approximately 80.7% pruning. This indicates that a substantially compressed CNN would be needed to match the parameter budget of the 3-filter MF-QCNN. Prior work on pruned deep-CNN-based channel estimation has investigated pruning as an accuracy–complexity trade-off rather than as a purely lossless parameter-reduction technique [42]. Therefore, compressing the CNN to a similar parameter level may lead to some NMSE degradation, although the extent of this degradation would depend on the pruning strategy and retraining procedure and would not necessarily reach the level of the single-filter QCNN. In contrast, the proposed MF-QCNN increases the quantum feature dimension by using multiple parallel PQC filters while keeping each filter shallow. These observations suggest that the proposed MF-QCNN can be viewed as a different compact-model design route from pruning a fully classical CNN.

Because the model directly outputs a structured complex-valued channel matrix, the same architecture may be adaptable to other RIS-assisted or two-hop channel estimation settings beyond the uplink scenario considered here. The filter count *F* can also be viewed as an adjustable design parameter for balancing estimation accuracy and quantum-resource usage, as suggested by the filter-sweep results in Section 5.

Regarding scalability to larger RIS-assisted systems, the number of qubits and the convolution–pooling depth required by each PQC filter are expected to grow with the system scale. For a system with *K* single-antenna users and an $N_{t}$-antenna BS, the direct real-valued embedding used in this work produces $2KN_{t}$ input features, corresponding to $2KN_{t}$ qubits under the same one-feature-per-qubit encoding strategy. Therefore, larger values of *K* or $N_{t}$ would increase the input dimension and may require deeper convolution–pooling structures and more quantum trainable parameters beyond the 182F+696 scaling reported in Section 5.

### Limitations and Future Work

The conclusions above should be interpreted in light of several limitations of this work. The evaluation relies on noiseless, idealized quantum-circuit simulation with parameter-shift-rule training. Under this idealized setting, the reported NMSE and sum-rate gains shown in Figure 9, Figure 10 and Figure 12 should be interpreted as an upper bound on the achievable performance rather than as a level directly attainable on near-term hardware. On physical NISQ devices, gate errors, decoherence, limited qubit connectivity, and the measurement (shot-sampling) overhead required to estimate the Pauli-*Z* expectation values would all tend to degrade the estimation accuracy, so the gains observed here are likely to be reduced when the model is executed on real quantum hardware.

Nevertheless, the proposed MF-QCNN is conceptually compatible with standard quantum error-mitigation techniques. Because the architecture relies only on Pauli-*Z* expectation values measured on four measurement qubits per filter, lightweight schemes such as measurement error mitigation could be applied directly to these measured features without altering the parallel-filter structure, and the shallow, fixed-depth design of each PQC filter keeps the circuit depth within a range that is comparatively amenable to such mitigation. A detailed evaluation of MF-QCNN under realistic noise models, together with measurement error mitigation and other quantum error-mitigation strategies, is left for future work.

In addition, the numerical study was conducted under a small-scale system configuration (K=2 users, $N_{t}=2$ BS antennas, and N=32 RIS elements), and although the expected qubit and parameter count growth with system scale is discussed above, the scalability of the proposed architecture to substantially larger deployments has not been numerically verified. Addressing these points through noisy-hardware validation, quantum error mitigation, larger-scale evaluation with more users and RIS elements, evaluation under more realistic wireless channel conditions, matched-budget comparisons with lightweight classical estimators, and hardware-aware PQC design aligned with current NISQ-device constraints is left for future work.

## 7. Conclusions

This paper proposed an MF-QCNN for cascaded channel estimation in RIS-assisted multi-user systems based on uplink pilot observations. The proposed architecture employs *F* independent PQC filters in parallel, rather than increasing the depth of a single quantum circuit. By combining multiple quantum feature representations extracted from the same embedded channel observation, the MF-QCNN benefits from a parallel-filter structure while maintaining a compact hybrid quantum–classical structure.

Numerical evaluations confirmed that the proposed MF-QCNN consistently outperforms the single-filter QCNN and the classical CNN and MLP baselines. At an SNR of approximately 20dB, the 3-filter MF-QCNN achieves an NMSE of approximately −25.7dB, corresponding to about 22.9dB, 8.1dB, and 4.6dB reductions compared with the single-filter QCNN, CNN, and MLP, respectively. Under ZF precoding, the 3-filter MF-QCNN improves the achievable sum rate by approximately 17.4% and 12.8% over the CNN and MLP baselines, respectively, at an SNR of approximately 30dB. It also uses only about 19.3% and 20.9% of the trainable parameters required by the CNN and MLP, respectively.

The filter-sweep analysis revealed a clear diminishing-return behavior as the number of PQC filters increases. The largest performance improvement occurs when the filter count increases from F=1 to F=2, whereas the gain from F=3 to F=4 is much smaller. This observation is important from the viewpoint of NISQ-era implementation, where additional PQC filters increase the number of quantum parameters, circuit evaluations, and parameter-shift gradient computations. Therefore, in the considered system configuration, the 3-filter MF-QCNN can be viewed as a balanced configuration that captures most of the NMSE and sum-rate gains while avoiding unnecessary quantum-resource overhead.

These results suggest that the proposed parallel multi-filter design offers a promising research direction for quantum-aided cascaded channel estimation in RIS-assisted B5G/6G systems.

## Figures and Tables

**Table 1:** Notation list.

Notation	Description
$N_{t}$	Number of BS antennas
*K*	Number of single-antenna users
*N*	Number of RIS reflecting elements
$d_{0}$	Reference distance
$d_{br}$	BS–RIS distance
$d_{ru,k}$	RIS–user distance for user *k*
$\tau_{br}$	BS–RIS path-loss exponent
$\tau_{ru}$	RIS–user path-loss exponent
$L_{br}$	BS–RIS path loss
$L_{ru,k}$	RIS–user path loss for user *k*
$H_{br}$	BS–RIS channel matrix
$H_{ru}$	RIS–user channel matrix
Φ	RIS phase-shift matrix
G	Cascaded channel matrix
$T_{p}$	Pilot sequence length
S	Uplink pilot matrix
R	Received pilot signal matrix at the BS
$\tilde{G}$	Pilot-processed noisy cascaded channel observation
N	AWGN matrix before pilot processing
Z	Effective noise matrix after pilot processing
$\sigma_{z}^{2}$	Noise variance

**Table 2:** Simulation parameter list.

Parameter	Value
Number of BS antennas ($N_{t}$)	2
Number of users (*K*)	2
Number of RIS elements (*N*)	32
Reference distance ($d_{0}$)	20 m
BS–RIS distance ($d_{br}$)	50 m
RIS–user distances ($d_{ru,k}$)	25 m, 30 m
BS–RIS path-loss exponent ($\tau_{br}$)	2.2
RIS–user path-loss exponent ($\tau_{ru}$)	2.8
Channel model	Rayleigh fading
Cascaded channel dataset	50,000 samples
Training/Validation/Test split	35,000/7500/7500 samples (70%/15%/15%)
Evaluation SNR range	−10 to 30 dB in 5 dB steps
Monte Carlo trials	1000
Optimizer	Adam
Learning rate (η)	$2\times 10^{-3}$
Training epochs	100
Number of qubits	8
Filter-sweep configurations	F=1 QCNN; F=2,3,4 MF-QCNN

**Table 3:** Complexity comparison of the MLP, CNN, QCNN, and MF-QCNN models.

Model	Trainable Parameters	Model Size	Circuit Evaluations		Simulated FLOPs
			Inference	Training	
MLP	5944	23.22 KB	—	—	11.5 K
CNN	6424	25.09 KB	—	—	28.7 K
QCNN (F=1)	878	3.43 KB	1	109	1.17 M
MF-QCNN (F=2)	1060	4.14 KB	2	218	2.34 M
MF-QCNN (F=3)	1242	4.85 KB	3	327	3.51 M
MF-QCNN (F=4)	1424	5.56 KB	4	436	4.68 M

## Data Availability

The original contributions presented in this study are included in the article. Further inquiries can be directed to the corresponding author.
